# Group decisions and individual differences: route fidelity predicts flight leadership in homing pigeons (*Columba livia*)

**DOI:** 10.1098/rsbl.2010.0627

**Published:** 2010-09-01

**Authors:** Robin Freeman, Richard Mann, Tim Guilford, Dora Biro

**Affiliations:** 1 Department of Zoology, University of Oxford, South Parks Road, Oxford OX1 3PS, UK; 2 Microsoft Research, Roger Needham Building, 7 JJ Thomson Avenue, Cambridge CB3 0FB, UK; 3 Matematiska Institutionen, Uppsala Universitet, Box 480, 751 06, Uppsala, Sweden

**Keywords:** navigation, homing pigeon, collective behaviour, decision-making, leadership

## Abstract

How social-living animals make collective decisions is currently the subject of intense scientific interest, with increasing focus on the role of individual variation within the group. Previously, we demonstrated that during paired flight in homing pigeons, a fully transitive leadership hierarchy emerges as birds are forced to choose between their own and their partner's habitual routes. This stable hierarchy suggests a role for individual differences mediating leadership decisions within homing pigeon pairs. What these differences are, however, has remained elusive. Using novel quantitative techniques to analyse habitual route structure, we show here that leadership can be predicted from prior route-following fidelity. Birds that are more faithful to their own route when homing alone are more likely to emerge as leaders when homing socially. We discuss how this fidelity may relate to the leadership phenomenon, and propose that leadership may emerge from the interplay between individual route confidence and the dynamics of paired flight.

## 1. Introduction

Until recently, the role of individual variation in group decision-making has been largely overlooked in favour of exploring the dynamics of homogeneous groups. Here, each individual is assumed to contribute to the group's decision with equal weight (e.g. [1])—a potentially problematic assumption given that many animal groups are composed of members differing in age, experience, skill, social rank and so forth. Recent studies have begun to explore the importance of individual variation in collective decision-making, demonstrating that individual properties can predict leading and following behaviour within group decision-making tasks (e.g. [2–7]; see also [8] for a review).

We recently demonstrated a transition from compromise to leadership during navigational decision-making by homing pigeons during paired flight [9]. Above a critical level of interindividual conflict (measured as the distance between two birds' individually established homing routes), pairs chose to fly along one of the birds' routes, rather than take an intermediate path. We noted that the resulting leadership hierarchy was *fully transitive* (in all cases, if bird A followed bird B and bird B followed bird C, then bird A would follow bird C), highlighting a role for individual variation during group decision-making. Surprisingly, this leadership phenomenon was not correlated with individual navigational efficiency, that is, birds with more efficient routes during solo flights did not tend to emerge as leaders when flying with a partner (but see [10] for larger flocks). Nevertheless, the existence of a stable hierarchy is indicative of idiosyncratic properties that mediate the formation of consistent and predictable leader–follower relationships within the group. We reasoned that, as the existence of recapitulated routes has demonstrated idiosyncrasy in birds' preferences during solo flight [11], the ability or motivation of individuals to accurately follow these routes may be a relevant individual property. Thus, we examine here the ability to predict leader–follower behaviour in paired flight using individual *route fidelity* in solo homing flights.

## 2. Material and methods

### (a) The dataset

We used data gathered in a previous study of 22 experienced homing pigeons, in which birds had formed habitual routes from three different sites: Greenhill Farm (distance and direction to home: 8.6 km, 197°, 12 subjects), Weston Wood (10.7 km, 221°, six subjects), and Church Hanborough (5.3 km, 129°, four subjects). Training consisted of a series of 20 repeated flights, during which subjects were always released singly and their paths were recorded using precision global positioning system loggers. For the present analysis, we use the last five training flights of each subject performed at its respective release site. This previously unpublished solo flight dataset thus consists of the training flights for the paired dataset presented in [9] and it allows us to characterize each subject in terms of its fidelity to an idiosyncratically preferred homing route.

Following training, birds at each site were randomly assigned a partner (trained from the same site), and released in pairs. Each bird was assigned to several different pairings, but only flew with any given partner once (see [9] for further details). For our present analysis, we selected only those 35 pairings (of a total of 48) in which the two birds remained together for the duration of the homing flight.

### (b) Data analysis

We examined the relationship between individual birds' performance during solo flights—in terms of the accuracy with which they recapitulated their established habitual routes during the final stages of training—and their propensity to emerge as ‘winners’ of pairwise interactions.

#### (i) Measuring individual route fidelity

To characterize the level of variability in a bird's habitual route, we first constructed a *mean path* based on the individual's final five solo flights during the training phase, then calculated the *variance* of the same five paths around every point of the mean trajectory.

Each solo homing flight was first normalized to 500 points using piecewise cubic-spline interpolation. The mean path was then constructed iteratively as follows. A set of 500 sequential points was created on a straight ‘thread’ between the release site and the home loft. At each iteration, each point was moved to the average position of its nearest neighbouring points on the five original tracks. To maintain an even distribution of the points along the thread, points that were more than 5 m from their neighbouring points were moved to their midpoint. Over a number of iterations (100 here), a sequence of points that lie at the average nearest neighbours of the original tracks is thus created and we call this the *mean path*. At each point along this mean path, the variance of the distances to the nearest neighbouring point on each original track was calculated, resulting in a distribution of route fidelity for each individual. We then considered a number of measures of this distribution, characterizing each individual bird by either the *mean route fidelity* over the whole track, the *peak route fidelity*: the minimum 5 per cent of the variance (25 locations along the entire length of the mean route), or the *initial route fidelity*: the first 5 per cent of the variance (the first 25 locations along the mean route). Peak route fidelity aims to capture the ability of an individual to recapitulate its route in regions where it can best do so; and initial route fidelity considers only those parts of the birds' routes that they are first likely to be in contact with following release as a pair (i.e. the early segment of flight during which the leadership contest is most probably decided). We also varied the percentages of the route considered in these analyses (1–20%) to examine the robustness of the predictions.

#### (ii) Determining winners and losers during paired flight

As in our previous work [9], the winner of any given pairing was determined based on the relative proximity that the two birds were able to maintain their respective habitual routes. We measured nearest-neighbour distances between each point on a bird's paired path and its own solo path flown immediately prior to the pairing. Within each pair, the bird with the smaller average nearest-neighbour distance between its paired and solo flight was declared the winner (leader) while its partner was designated the loser (follower).

## 3. Results

**Figure 1.**
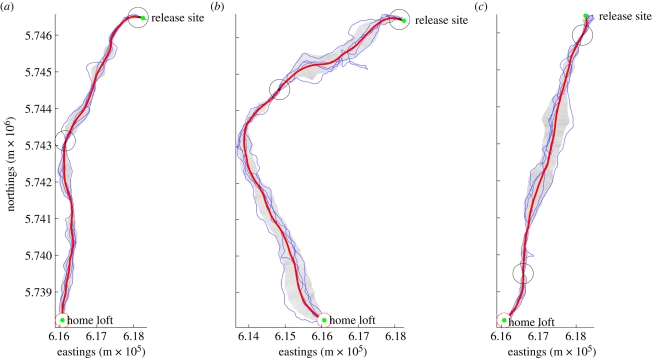
Three subjects' final five solo training flights (light blue), calculated mean path (bold red), variance around the mean path (grey band) and location of areas of peak route fidelity (black circles). Each peak fidelity region can contain multiple successive points, but for clarity only one circle is shown per region.

Figure 1 shows examples of individual birds' solo performance during their final five training flights, the iteratively constructed mean path and its variance over the course of the journey. The average of the set of point-by-point variances around the mean path provides a measure of the bird's fidelity to its habitual path. High fidelity (low variance) indicates a bird that is highly faithful to its mean path, while low fidelity corresponds to birds that recapitulate routes with relatively low accuracy. Each bird can thus be assigned a unique score; the range of mean fidelities was 85–282 m.

**Figure 2.**
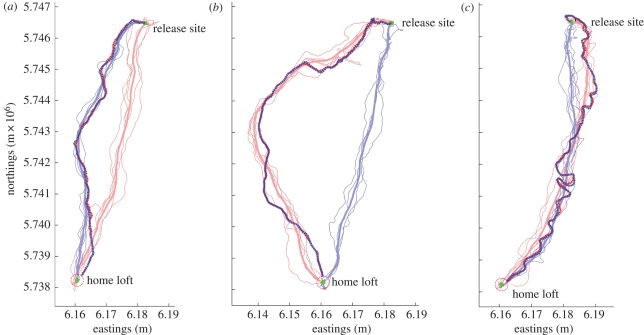
Examples of three different pairings. Each panel shows the training tracks (thin, solid lines), calculated mean paths (thick, solid lines) and paired tracks (thick, dashed bold line) for both birds of the pair (red and blue). Two correctly predicted pairings (*a* and *b*) and one incorrectly predicted pair (*c*) using the 5% peak fidelity measure are shown.

Across all three release sites, in 26 out of 35 cases the leader within a given pair corresponded to the bird with the higher *peak route fidelity* (figure 2). In other words, the minimal variance of an individual's habitual route was a significant predictor of leadership (*p* < 0.01, binomial test; note that as we are considering a specific property of each pair—the relative individual route variance—we consider pairings as independent events). At each release site, peak route fidelity successfully predicted leadership in roughly three-quarters of paired releases: Greenhill Farm: 15 of 20, Weston Wood: 8 of 11; Church Hanborough: 3 of 4. Mean route fidelity predicted only 23 out of 35 cases, and initial route fidelity predicted 24 out of 35. However, these results vary slightly with the proportion of the route being considered (see the electronic supplementary material, table S1).

Importantly, we note that when individuals are considered in relation to the leadership hierarchy presented in [9]—where birds that won all of their pairwise contests are ranked highest and those that always lost are ranked lowest—pairs that are not accurately predicted are usually only a single hierarchical level apart (using 5% peak fidelity, eight out of nine incorrectly predicted pairs were a single level apart). Thus, route fidelity best predicts those outcomes that are most clear-cut.

## 4. Discussion

We have previously shown that when pairs of homing pigeons have conflicting habitual routes, a transition from compromise to leadership occurs once the distance between the two birds' routes rises above a given threshold [9]. Here, we demonstrate that underlying properties of individual flight dynamics can, at least in part, predict the outcome of the leadership contest within these pairs. Using a novel technique to construct a mean spatial path and examining in detail birds' fidelity along it, we see that in a significant number of cases, peak route fidelity appears to influence the formation and control of leadership during paired flight. Peak fidelity is a better indicator of leadership than the mean fidelity over the full flight and may correspond to fidelity in the vicinity of a bird's memorized landmarks. This interpretation is consistent with the improvement in the predictive value of the peak route fidelity as smaller subsets of the flight path are considered. The smaller the eligible percentile of route fidelity the more probable this is to correspond solely to regions in the vicinity of landmarks.

This peak fidelity may be a property of the landmarks the birds have chosen to memorize, of the robustness of the memories themselves, or of the birds' motivation to use those memorized landmarks during homeward flight (perhaps similar to the *β* parameter modelled in [12]). Even if this characteristic is not evident at the point where the leadership decision is likely to have been made (see initial fidelity above), it is still the peak in propensity to fly faithfully to the route that appears to affect the decision—suggesting, perhaps, that the leader is more ‘confident’ about its own route, or less flexible in accepting to be drawn away from it. Similar results have recently been obtained in sticklebacks (*Gasterosteus aculeatus*), where ‘bold’ individuals (determined by their propensity to leave cover—potentially analogous to confidence/route fidelity here) were more likely to lead and, interestingly, enhanced the followership of their partners [6].

How this confidence—or indeed inflexibility—influences the decision whether to lead or follow remains an interesting open question. Considering two individuals that differ in their route fidelity, we may expect the individual with lower fidelity to be more willing to compromise—either owing to a lower level of confidence or to an increased tendency to be flexible in the route flown—eventually leading to abandonment of its route. It may, therefore, be that the leadership phenomenon is a property that emerges from the dynamics of individual route confidence/flexibility and paired flight. The overall importance of such individual differences for group outcomes are likely to be considerable, therefore future modelling and theoretical efforts may benefit from a better understanding of the nature and extent of individual variation in animal groups.
